# The Relationship between Pain Beliefs and Physical and Mental Health Outcome Measures in Chronic Low Back Pain: Direct and Indirect Effects

**DOI:** 10.3390/healthcare4030058

**Published:** 2016-08-19

**Authors:** Andrew Baird, David Sheffield

**Affiliations:** Centre for Psychological Research, Kedleston Road Campus, University of Derby, Derby DE22 1GB, UK; d.sheffield@derby.ac.uk

**Keywords:** low back pain, pain beliefs, disability, pain self-efficacy, anxiety, depression, locus of control

## Abstract

Low back pain remains a major health problem with huge societal cost. Biomedical models fail to explain the disability seen in response to reported back pain and therefore patients’ beliefs, cognitions and related behaviours have become a focus for both research and practice. This study used the Pain Beliefs Questionnaire and had two aims: To examine the extent to which pain beliefs are related to disability, anxiety and depression; and to assess whether those relationships are mediated by pain self-efficacy and locus of control. In a sample of 341 chronic low back pain patients, organic and psychological pain beliefs were related to disability, anxiety and depression. However, organic pain beliefs were more strongly related to disability and depression than psychological pain beliefs. Regression analyses revealed that these relationships were in part independent of pain self-efficacy and locus of control. Further, mediation analyses revealed indirect pathways involving self-efficacy and, to a lesser extent chance locus of control, between organic pain beliefs, on the one hand, and disability, anxiety and depression, on the other. In contrast, psychological pain beliefs were only directly related to disability, anxiety and depression. Although longitudinal data are needed to corroborate our findings, this study illustrates the importance of beliefs about the nature of pain and beliefs in one’s ability to cope with pain in determining both physical and mental health outcomes in chronic low back pain patients.

## 1. Introduction

Despite considerable attention, low back pain remains one of medicine’s most enigmatic problems, particularly in its chronic form. The majority of people will experience back pain at some point in their lives [1], but only a minority will receive a definitive diagnosis [2,3]. Most low back pain is largely non-specific in nature, but nevertheless has a societal cost greater than that for cancer, coronary artery disease and AIDS combined [4]. A systematic review by Dagenais, Caro and Haldeman [5] showed inconsistencies in the calculation of costs but found all studies to indicate back pain to be a substantial burden on society. In terms of healthcare costs, a recent study from the UK indicates that costs associated with chronic low back pain sufferers were twice that of matched controls [6]. The situation has been described in terms of an “epidemic” [7] and as a “20th Century medical disaster” [8] and is still perhaps one of the greatest examples of the failure of biomedical approaches. Biopsychosocial perspectives now dominate the literature, with psychosocial factors recognized as being fundamental in both assessment [9,10] and treatment/management [11,12]. Key to understanding the psychosocial influence is the consideration of individuals’ beliefs, cognitions and behaviours.

Therefore, addressing patients’ pain beliefs, cognitions and associated behaviours has become a major issue in pain management, particularly in chronic pain. Beliefs and associated behaviours have been associated with: The level of activity interference [13]; the frequency of pain behaviour [14]; the severity of pain experienced [15]; and levels of associated depression [16]. Two of the most important constructs in this area, which are driven by beliefs and which influence subsequent cognition and behaviour, are fear and catastrophising. These overlapping constructs impact upon vigilance to pain which can in turn also lead to increases in perceived pain severity [17]. A recent meta-analysis showed the relationship between fear and disability to be moderate to large in magnitude [18].

Ultimately, pain-related fear is more disabling than pain itself [19] as fear motivates avoidance behaviours [20,21]. In turn, avoidance behaviour affects activities of daily living and has a role in the transition from acute to chronic pain [22]. One of the key elements of fear is that of fear of further injury and re-injury [23], which can be a major barrier to recovery. In addition, expectancy beliefs are also a key factor in chronic pain. Ashari and Nicholas [24] showed pain self-efficacy beliefs to be an important determinant of pain behaviours and the disability associated with pain. More recently, Denison et al.’s [25] findings suggest that self-efficacy beliefs are even more important determinants of disability than fear avoidance beliefs in primary health care patients with musculoskeletal pain. Pain self-efficacy can mediate the relationship between clinical predictors and outcome measures. Pain self-efficacy mediates the relationship between pain severity and associated disability [26,27] and between pain related fear and disability [28]. It also mediates the relationship between fear and pain intensity [28] and between pain intensity and related depression [26]. Internal pain control can mediate reduction in levels of depression and pain behaviour following treatment [29].

Addressing problematic beliefs and related cognitions and behaviours can however, bring improvement in function [30]. Modern back pain management programmes are frequently grounded in the proven effectiveness of cognitive behavioural approaches [31,32,33] and exercise [34,35].

In the current study, participants were from a programme that explicitly addresses pain beliefs with participants [36], particularly the erroneous notion that ”hurt = harm” and ”more hurt = more harm”. Addressing these ”organic” pain beliefs has been shown to be associated with improvements in function following a rehabilitation programme [37] and the strength of these beliefs has been shown to clearly differentiate patients with chronic low back pain from the general population [38]. This study aims to assess the extent to which pain related physical and mental outcome measures can be explained by individuals’ pain beliefs as measured with the Pain Beliefs Questionnaire (PBQ) [39]. Furthermore, it aims to assess the extent to which the relationship between pain beliefs and physical and mental outcome measures is mediated by pain self-efficacy and locus of control.

## 2. Materials and Methods

### 2.1. Sample

The sample comprised 341 individuals who had been referred to the Nottingham Back Team programme located in Nottinghamshire, England. It is multi-disciplinary back pain management programme for individuals with chronic low back pain, delivered using 7 half day sessions on consecutive weeks. It is undertaken in community settings, i.e., utilising facilities at leisure centres as an alternative to requiring participants to visit a hospital site. The programme takes a cognitive behavioural approach and patients were informed that each session would incorporate, education, exercise, relaxation and discussions with a key worker (including goal setting). The sessions are delivered by a combination of physiotherapists, occupational therapists and nurses, with additional input from a clinical psychologist. The authors are independent of the programme and have no clinical role within it. Participants completed the standard battery of tests used within the programme together with the Pain Beliefs Questionnaire. Every patient that attended for assessment during the research period consented for their data to be used in the research, but not all responses were complete (n = 290). All data was gathered at assessment, before commencement of the programme.

### 2.2. Measures

The PBQ consists of 12 items representing two scales, which the authors described as “organic” (8 items) and “psychological” (4 items) [37]. One advantage of this questionnaire is that it is designed such that it is not necessary for the completer to be in pain or suffering from a specific condition (i.e., it is condition independent). The PBQ has previously been used successfully in research investigating chronic back pain [37,38,39,40] and with a UK general population [38]. On this occasion, the original 6 point (“always” to “never”) scale response was modified to a 5 point scale fit better with other questionnaires it was being used with, as part of the broader research [38]. The five items were “All of the time”, “Most of the time”, “Some of the time”, “A little of the time”, “None of the time”. In their study with patients experiencing chronic low back pain, Walsh and Radcliffe also describe using a 5-point scale [37]. The PBQ organic and psychological sub-scales are scored using the sum of the items for each scale respectively.

The Roland-Morris Disability Questionnaire (RDQ) [41] is one of the most widely used measures of back pain related disability and was originally derived from the Sickness Impact Profile (SIP). Twenty-four items that related to physical functions potentially affected by back pain were selected from the SIP. Each item was then qualified with the phrase “because of my back pain” to differentiate back pain disability from disability due to other causes [42]. Patients are asked to indicate which of the series of statements applies to them on the day of completing the questionnaire. The RDQ provides a single score which is calculated by summing the number of items selected. The items are not weighted in any way. The scores therefore range from 0 (no disability) to 24 (maximum disability). The RDQ is short, easy to understand and simple to complete [42].

The Pain Self-efficacy Questionnaire [43] is a ten item questionnaire, with each item assessed on a scale from 0 to 6 where 0 indicates no confidence and 6 indicates complete confidence. The 10 items are designed to cover a range of factors relating to activities of daily living. The items are not weighted and the questionnaire provides a single scale. The scores therefore range from 0, indicating no confidence in carrying out activities of daily living, to 60, indicating complete confidence in carrying out normal activities of daily living.

The Hospital Anxiety and Depression Scale (HADS) [44] is a 14 item screening tool comprising two seven item scales—anxiety and depression. To ensure a focus on anxiety and depression, symptoms which could also be associated with somatic disorders were excluded as were those indicative of more complex/serious psychiatric conditions. It is a widely used instrument in both clinical practice and research. A review by Bjellanda et al. [45] identified over seven hundred papers reporting use of HADS. It has been found to have good psychometric properties and performed well in assessing the general severity and ‘caseness’ of both anxiety disorders and depression. It has been widely used in trials relating to back pain including the large STarT Back trial [46].

The Multi-Dimensional Health Locus of Control Questionnaire [47] is an 18 item questionnaire comprising 3 scales—internal, chance and powerful others. The measure has been used for several decades and the scales are considered reliable and valid [48]. It is a general measure; not specific to particular symptoms or conditions. The measure has been used in back pain research in the past [49] and it was used during the development of the Pain Beliefs Questionnaire [39].

### 2.3. Analytic Strategy and Scoring

**Figure 1:**
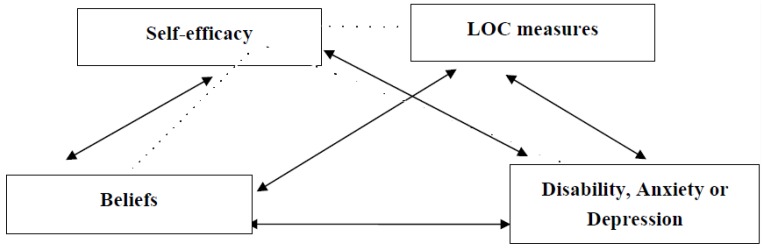
Conceptual Model of Bootstrapped Mediation Model.

Initial analyses focused on examining differences in age and gender between those who completed the questionnaires and those that did not. Gender data was not available from one completer and one non-completer of all questionnaires. Then attention focused on correlations between beliefs, self-efficacy, locus of control, and depression, anxiety and disability. Next, regression analysis was used to examine the independent contributions that beliefs, self-efficacy, and locus of control variables make to the prediction of depression, anxiety and disability. Finally, a bootstrapped mediation model tested the conceptual model outlined in Figure 1. For each belief-mental or physical outcome relationship hypotheses were tested simultaneously using the “Process” macro for SPSS [50], with 5,000 bootstrapping re-samples and bias-corrected 95% confidence intervals (CIs) for each indirect effect. In bootstrapping analyses, bias corrected CIs that do not contain 0 signify a significant indirect effect [51,52]. Direct effects estimate how much two cases differing on the independent variable (organic or psychological belief) also differ on the dependent variable (disability, anxiety, or depression) independent of the effect of the mediator variables (self-efficacy and locus of control variables) on the dependent variable. Total effects are the sum of the indirect and direct effects of the independent variables on the dependent variable [50]. Alternate models were tested and these are presented in the Appendix. Analysis was conducted using IBM SPSS 22 for Windows with an alpha = .05. No participants were excluded from the analysis.

### 2.4. Ethics

Ethical approval for the study was provided through the Nottingham Back Team via National Health Service processes. Participants received both written and verbal explanation during their assessment before informed consent was sought for their data to be used for the purposes of this research.

## 3. Results

### 3.1. Gender and Age Differences

Individuals who completed all questionnaires were significantly older (mean ± S.D. = 45.35 ± 13.14 vs. 48.02 ± 13.61 years) and more likely to be men (43.8% vs. 31.8%) than those who did not, *p* < 0.05.

### 3.2. Demographics

Examination of the mean and standard deviations suggested that participants were similar to previous low back pain samples [46]; see Table 1.

### 3.3. Bivariate Correlations

There were no gender differences in any predictor or outcome measures, *p* > 0.1. Age was negatively related to anxiety, *r* = −0.15, *p* < 0.01, and internal locus of control, *r* = −0.13, *p* < 0.05. Accordingly, age was entered in subsequent regression analyses. Pearson’s correlations revealed that disability, anxiety and depression were strongly inter-related but as the proportion of shared variance was less than 50% regression models with each as an outcome variable were calculated. Disability was strongly related to organic pain beliefs and self-efficacy, and more weakly, but significantly, related to psychological pain beliefs and locus of control measures. Anxiety was related to organic and psychological pain beliefs and self-efficacy, and more weakly, but significantly, related to internal and other locus of control measures; it was unrelated to chance locus of control. Depression was strongly related to organic pain beliefs and self-efficacy and more weakly related to psychological pain beliefs and locus of control measures. Fisher transformations revealed that organic pain beliefs were more strongly related to disability and depression than psychological pain beliefs (*p* < 0.05); correlations between organic and psychological pain beliefs and anxiety were not different. Organic pain beliefs were strongly related to self-efficacy and more weakly related to locus of control measures. Psychological pain beliefs were related to internal locus of control, but were not related to self-efficacy or chance and other locus of control (see Table 1).

### 3.4. Regression Analyses: Disability

After testing for multi-collinearity (all VIF < 2; all tolerances > 0.5), analysis revealed that the regression model was significant (*F*(7, 333) = 56.82, *p* < 0.001), with 54% of the variance in the outcome being explained by the predictors (*R^2^* = 0.544, adjusted *R^2^* = 0.535). There were significant positive relationships between organic pain beliefs and disability and between psychological pain beliefs and disability, and a significant negative relationship between pain self-efficacy and disability; there were no other significant relationships (see Table 2).

Results of the mediation analyses indicated that there was a significant indirect effect of organic pain beliefs on disability through self-efficacy, *b* = −0.23, *p* < 0.01, BCa CI (−0.27, −0.19), which explained 36% of the total effect. In contrast, there were no significant indirect effects of organic pain beliefs on disability through LOC measures. The direct effect of organic pain beliefs on disability was also significant, *b* = 0.25, *t* = 4.79, *p* < 0.0001. In contrast, there was no significant indirect effect of organic pain beliefs on disability through LOC measures. For psychological pain beliefs on disability, there were no significant indirect effects through self-efficacy or LOC measures. The direct effect of psychological pain beliefs on disability was significant, *b* = 0.26, *t* = 4.35, *p* < 0.0001. Analyses with belief and locus of control measures as mediators of the self-efficacy-disability relationship were also examined. These suggest that the model is less parsimonious than the one proposed; these are presented in the Appendix.

### 3.5. Regression Analyses: Anxiety

The regression model was significant (*F*(7, 333) = 25.39, *p* < 0.001), with 35% of the variance in the outcome being explained by the predictors (*R^2^* = 0.348, adjusted *R^2^* = 0.334). There were significant positive relationships between psychological pain beliefs and anxiety and between chance locus of control and anxiety, and significant negative relationships between age and anxiety and pain self-efficacy and anxiety; there were no other significant relationships (see Table 2).

Results of the mediation analyses indicated that there was a significant indirect effect of organic pain beliefs on anxiety through self-efficacy, *b* = −0.11, *p* < 0.0001, BCa CI (−0.15, −0.07), which explained 18% of the total effect, and through chance locus of control, *b* = 0.19, *p* < 0.0001, BCa CI (0.11, 0.27), which explained 5% of the total effect. There were no significant indirect effects of organic pain beliefs on anxiety through other LOC measures and there was no direct effect of organic pain beliefs on anxiety, *b* = 0.05, *t* = 1.06, *p* = 0.29. For psychological pain beliefs on anxiety, there were no significant indirect effects through self-efficacy or LOC measures. The direct effect of psychological pain beliefs on anxiety was significant, *b* = 0.26, *t* = 4.70, *p* < 0.0001.

### 3.6. Regression Analyses: Depression

The regression model was significant (*F*(7, 333) = 52.23, *p* < 0.001), with 52% of the variance in the outcome being explained by the predictors (*R^2^* = 0.523, adjusted *R^2^* = 0.513). There were significant positive relationships between organic pain beliefs and depression, psychological pain beliefs and depression, and between chance locus of control and depression, and a significant negative relationship between pain self-efficacy and depression; there were no other significant relationships (see Table 2).

Results of the mediation analyses indicated that there was a significant indirect effect of organic pain beliefs on depression through self-efficacy, *b* = −0.17, *p* < 0.0001, BCa CI (−0.19, −0.14), which explained 27% of the total effect, and through chance locus of control, *b* = 0.08, *p* < 0.005, BCa CI (−0.02, 0.13), which explained 2% of the total effect. There were no significant indirect effects of organic pain beliefs on depression through other LOC measures. There was a direct effect of organic pain beliefs on depression, *b* = 0.07, *t* = 2.20, *p* = 0.03. For psychological pain beliefs on depression, there were no significant indirect effects through self-efficacy or LOC measures. The direct effect of psychological pain beliefs on depression was significant, *b* = 0.10, *t* = 2.42, *p* = 0.01.

## 4. Discussion

This study had two aims: To examine the extent to which pain beliefs are related to disability, anxiety and depression; and to assess whether those relationships are mediated by pain self-efficacy and locus of control. In a sample of 341 low back pain patients, organic and psychological pain beliefs were related to disability, anxiety and depression. However, organic pain beliefs were more strongly related to disability and depression than psychological pain beliefs. Regression analyses revealed that these relationships were in part independent of pain self-efficacy and locus of control. Further, mediation analyses revealed indirect pathways involving self-efficacy and, to a lesser extent chance locus of control, between organic pain beliefs, on the one hand, and disability, anxiety and depression, on the other. In contrast, psychological pain beliefs were only directly related to disability, anxiety and depression.

The gender split of the 341 participants included in the study was comparable with other studies of chronic low back pain [46,53]. Consideration of the descriptive statistics for the commonly taken measures—RMDQ, PSEQ and HADS—shows a pattern fairly typical of chronic low back pain patients. A mean disability of 9.6 is similar to that found by Hill et al. within the STarT Back trial [46] (9.8), though slightly higher than in the large study by Foster et al. [53] (8.6). The PSEQ mean of 34.1 was similar to that found by Foster et al. [53] (37.8). This is higher than reported by Nicholas [54] (25.8), but lower than reported by Costa et al. [55] (44.4) in chronic low back pain patients, so it appears to be in the range reported for chronic low back pain patients. A mean anxiety score of 8.2 was again similar to that reported by Foster et al. [53] (8.3) albeit slightly higher than that reported by Hill et al. [46] (7.5). Depression scores were lower than anxiety scores as is consistent within the literature and the mean of 6.0 is in line with that reported by Foster et al. [53] (6.5) and Hill et al. [46] (5.9). Overall, therefore this could be said to be an ‘unremarkable’ sample of chronic low back pain patients.

Organic pain beliefs deal with both the perceived cause of an individual’s pain (“hurt = harm”) and its management (issues of control and exercise/activity). As such, they could be considered a measure of ‘biomedical thinking’ [38]. These organic beliefs are associated with the outcome measures of disability, anxiety and depression. Higher organic pain beliefs are associated with higher levels of disability, depression and anxiety. On its own it could interpreted to be a good predictor of these outcomes, but mediation analyses show that much of the effect (all in the case of anxiety) is indirect through self-efficacy and to a lesser extent chance locus of control. The cross-sectional nature of the data is such that different mediation models could be described, but the defined model is in keeping with the literature on pain self-efficacy [26,27,28] and was the most parsimonious one (see appendix).

Psychological pain beliefs are concerned with the impact of anxiety, depression, attention to pain and the issue of relaxation. These beliefs are also associated with the outcome measures, but the strength of the belief was much lower than for the organic beliefs. Higher levels of psychological pain beliefs are associated with higher levels of disability, depression and anxiety, but the relationship is relatively weak. Moreover, the mediation analyses showed that none of the effects were indirect through self-efficacy or locus of control measures. Thus, psychological pain beliefs are directly related to disability, depression and anxiety. Moreover, they appear to relate to markedly different constructs than other commonly used belief measures, such as fear avoidance beliefs, which like organic beliefs are mediated by self-efficacy [28]. Baird and Haslam [38] found that psychological beliefs differentiated between chronic low back pain patients and a non-clinical sample, but unlike with organic beliefs, there was no difference within the non-clinical sample between those who experienced frequent pain and those who did not. It was suggested that this may indicate that psychological pain beliefs are uniquely influenced by chronicity and not simply the presence of pain.

The PBQ has been used successfully with chronic low back pain samples [37,38,40] and this study supports the value of the measure. The scales illustrate both direct and mediated effects on key physical and mental health outcome measures. The measure has utility in both research and practice as it taps into clinically relevant beliefs that are amenable to change and are the focus of many rehabilitation programmes. Indeed, efforts to reduce the strength of these beliefs about the origins, nature and treatment of pain, particularly the ‘biomedical’ beliefs, should yield positive results in chronic low back pain rehabilitation [37,40].

This study supports the view that self-efficacy is an important predictor of disability [24,25,53]. It has a strong relationship with disability in bivariate analysis and its importance as an independent predictor of disability is confirmed in the regression models. Self-efficacy also has a strong association with mental health outcome measures, particularly depression. Further, the findings support previous studies indicating a role for self-efficacy as mediator between beliefs and outcomes [26,27,28]. Although Schiphorst Preuper et al. [56] questioned the impact of psychological variables, including self-efficacy, in a study in which psychological variables explained only 19% of the variance in self-reported disability, it was noticeable that the research used a general measure of self-efficacy and not a pain-specific measure as used in the current study and in previous studies mentioned above [24,28,53]. Pain self-efficacy may represent one of the most influential and most valuable psychological constructs in chronic low back pain [53] and it may be wise to measure this construct in both practice and research. These beliefs may be the target of clinical intervention via cognitive-behavioural, exercise-based programmes using both education and graded exposure.

The Multidimensional Health Locus of Control (MHLC) measure is a generic measure rather than a pain-specific questionnaire like the other scales used within the study. As such it may lack a degree of sensitivity with this patient population. The MHLC was used in the development of the Pain Beliefs Questionnaire [39] but it is noticeable that the correlations found in this study are weaker than those found by Edwards et al. [39]. The PBQ developers found no relationship between internal locus of control and organic pain beliefs, but this study indicated a negative relationship, albeit a relatively weak one. The regression analyses in this study illustrate that internal and powerful other locus of control are not predictors of any of the outcomes. Chance locus of control does however predict anxiety and, to a lesser extent, depression. Mediation analyses showed that there was some effect of organic pain beliefs on anxiety and depression which was indirect, through chance locus of control. Overall MHLC appears to have limited utility in relation to chronic low back pain.

This study utilises a suitably large sample whose characteristics indicate it to be a fairly typical chronic low back pain population. However, there were differences between questionnaire battery completers and non-completers. Failure to fully complete the questionnaire battery was perceived to be a consequence of time pressures during the assessment process. The difference in age (mean of 45 v 48 years) was not large however, given the nature of the sample. It was surprising that a higher proportion of men completed the battery than did not. Given the time pressure at initial assessment, men, who tend to be less conscientious than women [57], may have attempted the battery more quickly and so completed it.

Finally, the study is cross-sectional so causal inferences cannot be made from this data. The mediation models tested do reflect the literature, but it must be recognized that alternative models could be produced which could also fit with the available data, for example beliefs may mediate the relationships observed between self-efficacy and outcomes however this was a less good fit. Alternatively, unmeasured variables such as pain catastrophizing, may play a role in these relationships. Future longitudinal studies using the Pain Beliefs Questionnaire could further assess the usefulness of the measure in predicting and explaining variations in disability, anxiety and depression following low back pain rehabilitation programmes. In addition, controlled trials would help provide causal information about the role of beliefs in pain rehabilitation.

## 5. Conclusions

Overall, this study illustrates the importance of beliefs about the nature of pain and beliefs in one’s ability to cope with pain in determining both physical and mental health outcomes amongst chronic low back pain patients. The pain beliefs questionnaire is a simple measure that is independent of condition or the presence of pain, but nevertheless provides two scales that are useful in predicting disability and mental health outcomes. Pain self-efficacy is an excellent predictor of physical and mental pain-related outcomes and can act indirectly in the relationship between organic (‘biomedical’) beliefs about pain and disability, anxiety and depression.

## Figures and Tables

**Table 1:** Bivariate correlations and Means (SDs).

Variables	2	3	4	5	6	7	8	9	Mean (SD)
1. Disability	0.41 ***	0.57 ***	0.56 ***	0.21 ***	−0.70 ***	−0.19 ***	0.14 *	0.12 *	9.58 (5.34)
2. Anxiety	--	0.58 ***	0.34 ***	0.26 ***	−0.45 ***	−0.03	0.35 ***	0.21 ***	8.22 (3.97)
3. Depression	0.58 ***	--	0.50 ***	0.15 **	−0.70 ***	−0.13 **	0.26 ***	0.17 **	5.96 (3.54)
4. Organic Beliefs	0.34 ***	0.50 ***	--	0.13 **	−0.59 ***	−0.22 ***	0.27 ***	0.18 **	25.91 (4.90)
5. Psychological Beliefs	0.26 ***	0.15 **	0.13 **	--	−0.08	0.17 **	0.00	0.02	11.28 (3.50)
6. Self-efficacy	0.45 ***	−0.70 ***	−0.59 ***	−0.08	--	0.22 ***	−0.19 ***	−0.19 ***	34.09 (13.39)
7. LOC—Internal	−0.03	−0.13 **	−0.22 ***	0.17 **	0.22 ***	--	−0.01	0.06	26.58 (4.75)
8. LOC—Chance	0.35 ***	0.26 ***	0.27 ***	0.00	−0.19 ***	−0.01	--	0.39 ***	19.02 (5.27)
9. LOC—Other	0.21 ***	0.17 **	0.18 **	0.02	−0.19 ***	0.06	0.39 ***	--	20.07 (6.23)

LOC: locus of control; * *p* < 0.05; ** *p* < 0.01; *** *p* < 0.001; -- There is perfect correlation between the same variable/s.

**Table 2:** Beta (standard deviation) and *t*-values for Regression Models.

Variables	Disability Beta (SD)	*T*	Anxiety	*t*	Depression	*t*
Age	0.011 (0.016)	0.71	−0.046 (0.014)	−3.24 **	−0.014 (0.011)	−1.33
Organic Beliefs	0.222 (0.052)	4.26 ***	0.035 (0.046)	0.76	0.071 (0.035)	2.01 *
Psychological Beliefs	0.229 (0.058)	3.94 ***	0.259 (0.052)	5.02 ***	0.086 (0.039)	2.19 *
Self-efficacy	−0.225 (0.019)	−12.31 ***	−0.103 (0.017)	−6.24 ***	−0.163 (0.013)	−12.93 ***
LOC—Internal	−0.041 (0.044)	−0.93	−0.003 (0.040)	−0.07	0.002 (0.030)	0.07
LOC—Chance	−0.015 (0.043)	−0.34	0.170 (0.038)	4.47 ***	0.074 (0.029)	2.55 *
LOC—Other	−0.021 (0.036)	−0.57	0.048 (0.032)	1.49	−0.001 (0.024)	−0.06

* *p* < 0.05; ** *p* < 0.01; *** *p* < 0.001.

## Appendix

The main body of the paper describes the most parsimonious model, but further analyses were undertaken to consider alternatives in relation to disability, anxiety and depression as described below:

Disability: In order to test the possibility that a third unmeasured variable may account for these relationships, further mediation analysis was conducted, with belief and LOC measures as mediators of the self-efficacy-disability relationship. There was a significant indirect effect of self-efficacy on disability through organic pain beliefs, *b* = −0.23, *p* < 0.0001, BCa CI (−0.07, −0.03), which explained 5% of the total effect. The direct effect of self-efficacy on disability was also significant, *b* = −0.22, *t* = −12.13, *p* < 0.0001.

Anxiety: In order to test the possibility that a third unmeasured variable may account for these relationships, further mediation analysis was again conducted, with belief and LOC measures as mediators of the self-efficacy-anxiety relationship. There was a significant indirect effect of self-efficacy on anxiety through chance LOC, *b* = −0.20, *p* < 0.0001, BCa CI (−0.03, −0.01), which explained 1% of the total effect. The direct effect of self-efficacy on anxiety was also significant, *b* = −0.11, *t* = −6.50, *p* < 0.0001.

Depression: Again, to test the possibility that a third unmeasured variable may account for these relationships, further mediation analysis was conducted, with belief and LOC measures as mediators of the self-efficacy-depression relationship. There was a significant indirect effect of self-efficacy on depression through chance LOC, *b* = −0.08, *p* < 0.005, BCa CI (−0.01, 0.00), which explained 1% of the total effect. The direct effect of self-efficacy on depression was also significant, *b* = −0.16, *t* = −13.11, *p* < 0.0001.

## References

[1] Balague F., Manion A.F., Pellise F., Cedraschi C. (2012). Non-specific low back pain. Lancet.

[2] Snook S. (2004). Work-related low back pain: Secondary intervention. J. Electromyogr. Kinesiol..

[3] Deyo R., Weinstein J. (2001). Low back pain. N. Engl. J. Med..

[4] Thomsen A., Sorenson J., Sjorgen P., Eriksen J. (2002). Chronic non-malignant pain patients and health economic consequences. Eur. J. Pain.

[5] Dagenais G., Caro J., Haldeman S. (2008). A systematic review of low back pain cost of illness studies in the United States and internationally. Spine J..

[6] Hong J., Reed C., Novick D., Happich M. (2013). Costs associated with treatment of chronic low back pain: An analysis of the UK General Practice Research Database. Spine.

[7] Lidgren L. (2003). The Bone and Joint Decade and the global economic and healthcare burden of musculoskeletal disease. J. Rheumatol..

[8] Waddell G. (2004). The Back Pain Revolution.

[9] Waddell G., Burton A.K. (2001). Occupational health guidelines for the management of low back pain at work: Evidence review. Occup. Med. (Lond.).

[10] Main C.J., George S.Z. (2011). Psychologically informed practice for management of low back pain: Future directions in practice and research. Phys. Ther..

[11] Burton A.K., Balague F., Cardon G., Eriksen H.R., Henrotin Y., Lahad A., Leclerc A., Müller G., van der Beek A.J. (2006). Chapter 2. European guidelines for prevention in low back pain. Eur. Spine J..

[12] Chou R., Qaseem A., Snow V., Casey D., Cross J.T., Shekelle P., Owens D. (2007). Diagnosis and treatment of low back pain: A joint clinical practice guideline from the American College of Physicians and the American Pain Society. Ann. Intern. Med..

[13] Turner J., Dworkin S., Mancl L., Huggins K., Truelove E. (2001). The roles of beliefs, catastrophizing, and coping in the functioning of patients with temporomandibular disorders. Pain.

[14] Jensen M., Romano J., Turner J., Good A., Wald L. (1999). Patient beliefs predict patient functioning: Further support for a cognitive-behavioural model of chronic pain. Pain.

[15] Vowle K.E., Gross R.T. (2003). Work-related beliefs about injury and physical capability for work in individuals with chronic pain. Pain.

[16] Turner J., Jensen M., Romano J. (2000). Do beliefs, coping, and catastrophizing independently predict functioning in patients with chronic pain?. Pain.

[17] Goubert L., Crombez G., Van Damme S. (2004). The role of neuroticism, pain catastrophizing and pain-related fear in vigilance to pain: A structural equations approach. Pain.

[18] Zale E.L., Lange K.L., Fields S.A., Ditre J.W. (2013). The relation between pain-related fear and disability: A meta-analysis. J. Pain.

[19] Crombez G., Vlaeyen J.W., Heuts P., Lysens R. (1999). Pain-related fear is more disabling than pain itself: Evidence on the role of pain-related fear in chronic back pain disability. Pain.

[20] Vlaeyen J.W., Linton S.J. (2000). Fear-avoidance and its consequences in chronic musculoskeletal pain: A state of the art. Pain.

[21] Pfingsten M., Leibing E., Harter W., Kroner-Herwig B., Hempel D., Kronshage U., Hildebrandt J. (2001). Fear-avoidance behavior and anticipation of pain in patients with chronic low back pain: A randomized controlled study. Pain Med..

[22] Buer N., Linton S.J. (2002). Fear-avoidance beliefs and catastrophizing: Occurrence and risk factor in back pain and ADL in the general population. Pain.

[23] Shaw W.S., Main C.J., Johnston V. (2011). Addressing occupational factors in the management of low back pain: Implications for physical therapist practice. Phys. Ther..

[24] Ashari A., Nicholas M.K. (2001). Pain self-efficacy beliefs and pain behaviour. A prospective study. Pain.

[25] Denison E., Asenlof P., Lindberg P. (2004). Self-efficacy, fear avoidance, and pain intensity as predictors of disability in subacute and chronic musculoskeletal pain patients in primary health care. Pain.

[26] Amstein P., Caudill M., Mandler C., Norris A., Beasley R. (1999). Self-efficacy as a mediator of the relationship between pain intensity, disability and depression in chronic pain patients. Pain.

[27] Arnstein P. (2000). The mediation of disability by self-efficacy in different samples of chronic pain patients. Disabil. Rehabil..

[28] Woby S., Urmston M., Watson P. (2007). Self-efficacy mediates the relation between pain-related fear and outcome in chronic low back pain patients. Eur. J. Pain.

[29] Spinhoven P., ter Kuile A., Mansfeld M.H., den Ouden D.J., Vlaeyen J.W.S. (2004). Catastrophizing and internal pain control as mediators of outcome in the multidisciplinary treatment of chronic low back pain. Eur. J. Pain.

[30] Woby S., Watson P., Roach N., Urmston M. (2004). Are changes in fear-avoidance beliefs, catastrophizing, and appraisals of control, predictive of changes in chronic low back pain and disability?. Eur. J. Pain.

[31] Morley S., Eccleston C., Williams A. (1999). Systematic review and meta-analysis of randomized controlled trials of cognitive behaviour therapy and behaviour therapy for chronic pain in adults, excluding headache. Pain.

[32] van Tulder M., Ostelo R., Vlaeyen J., Linton S., Morley S., Assendelft W. (2000). Behavioral treatment for chronic low back pain: A systematic review within the framework of the Cochrane Back Review Group. Spine.

[33] Linton S.J., Ryberg M. (2001). A cognitive-behavioral group intervention as prevention for persistent neck and back pain in a non-patient population: A randomized controlled trial. Pain.

[34] Abenhaim L., Rossignol M., Valat J.P., Nordin M., Avouac B., Blotman F., Charlot J., Dreiser R., Legrand E., Rozenberg S. (2000). The role of activity in the therapeutic management of back pain. Report of the International Paris Task Force on Back Pain. Spine.

[35] Liddle S., Baxter G., Gracey J. (2004). Exercise and chronic low back pain: What works?. Pain.

[36] Baird A., Worral L., Haslam C., Haslam R. (2008). Evaluation of a multi-disciplinary back pain rehabilitation programme––Individual and group perspectives. Qual. Life Res..

[37] Walsh D.A., Radcliffe J.C. (2002). Pain beliefs and perceived physical disability of patients with chronic low back pain. Pain.

[38] Baird A.J., Haslam R.A. (2013). Exploring differences in pain beliefs within and between a large nonclinical (workplace) population and a clinical (chronic low back pain) population using the pain beliefs questionnaire. Phys. Ther..

[39] Edwards L., Pearce S., Turner-Stokes L., Jones A. (1992). The Pain Beliefs Questionnaire: An investigation of beliefs in the causes and consequences of pain. Pain.

[40] Sloan T., Gupta R., Zhang W., Walsh D. (2008). Beliefs about the causes and consequences of pain in patients with chronic inflammatory or non-inflammatory low back pain and in pain-free individuals. Spine.

[41] Roland M., Morris R. (1983). A study of the natural history of low back pain. Part 1: Development of a reliable and sensitive measure of disability in low-back pain. Spine.

[42] Roland M., Fairbank J. (2000). The Roland–Morris Disability questionnaire and the oswestry disability questionnaire. Spine.

[43] Nicholas M.K. Self-efficacy and Chronic Pain. Proceedings of the Annual Conference of the British Psychological Society.

[44] Zigmond A.S., Snaith R.P. (1983). The Hospital Anxiety and Depression Scale. Acta. Psychiatr. Scand..

[45] Bjellanda I., Dahlb A.A., Tangen Haugc T., Neckelmann D. (2002). The validity of the Hospital Anxiety and Depression Scale: An updated literature review. J. Psychosom. Res..

[46] Hill J.C., Whitehurst D.G.T., Lewis M., Dunn K.M., Foster N.E., Konstantinou K., Main C.J., Mason E., Somerville S., Sowden G., Vohora K., Hay E.M. (2011). Comparison of stratified primary care management for low back pain with current best practice (STarT Back): A randomised controlled trial. Lancet.

[47] Wallston K.A., Wallston B.S., DeVellis R. (1978). Development of the multidimensional health locus of control (MHLC) scales. Health Educ. Monogr..

[48] Wallston K.A. (2005). The Validity of the Multidimensional Health Locus of Control Scales. J. Health Psychol..

[49] Harkapaa K., Jarvikovski A., Mellin G., Hurri H., Luoma J. (1991). Health locus of control beliefs and psychological distress as predictors for treatmeant outcome in low-back pain patients: Results of a 3-month follow-up of a controlled intervention study. Pain.

[50] Hayes A.F. PROCESS: A versatile computational tool for observed variable mediation, moderation, and conditional process modelling. http://afhayes.com/public/process2012.pdf.

[51] Preacher K.J., Hayes A.F. (2004). SPSS and SAS procedures for estimating indirect effects in simple mediation models. Behav. Res. Methods Instrum. Comput..

[52] Preacher K.J., Hayes A.F. (2008). Asymptotic and resampling strategies for assessing and comparing indirect effects in multiple mediator models. Behav. Res. Methods.

[53] Foster N.E., Thomas E., Bishop A., Dunn K.M., Main C.J. (2010). Distinctiveness of psychological barriers to recovery in low back pain patients in primary care. Pain.

[54] Nicholas M.K. (2007). The pain self-efficacy questionnaire: Taking pain into account. Eur. J. Pain.

[55] Costa L.D.C.M., Maher C.G., McAuley J.H., Hancock M.J. (2011). Self-efficacy is more important than fear of movement in mediating the relationship between pain and disability in chronic low back pain. Eur. J. Pain.

[56] Schiphorst Preuper H.R., Reneman M.F., Boonstra A.M., Dijkstra P.U., Versteegen G.J., Geertzen J.H.B., Brouwer S. (2008). Relationship between psychological factors and performance-based and self-reported disability in chronic low back pain. Eur. Spine J..

[57] Vianello M., Schnabel C., Sriram N., Nosek B. (2013). Gender differences in implicit and explicit personality traits. Pers. Individ. Dif..

